# Ytterbium(III) Tricyanomethanides with Sodium and Potassium: Similarities and Differences Between NaYb[C(CN)_3_]_4_ and KYb[C(CN)_3_]_4_

**DOI:** 10.3390/molecules30214284

**Published:** 2025-11-04

**Authors:** Ralf J. C. Locke, Giuseppe Montana, Robert U. Stelzer, Anahita I. A. Emminghaus, Falk Lissner, Olaf Reckeweg, Thomas Schleid, Claudia Wickleder

**Affiliations:** 1 Institute for Inorganic Chemistry, University of Stuttgart, Pfaffenwaldring 55, D-70569 Stuttgart, Germanylissner@iac.uni-stuttgart.de (F.L.); o.reckeweg@gmx.de (O.R.); 2 Inorganic Chemistry, Department of Chemistry and Biology, University of Siegen, Adolf-Reichwein-Straße 2, D-57068 Siegen, Germany

**Keywords:** alkali and rare-earth metals, tricyanomethanides, structure elucidation, Raman spectra, synthesis

## Abstract

Metathesis reactions of Ag[C(CN)_3_] with anhydrous YbCl_3_ dissolved in water combined with stoichiometric amounts of the alkali-metal salts *A*[C(CN)_3_] (*A* = Na or K) yield the non-isotypic tetragonal compounds NaYb[C(CN)_3_]_4_ (*P*4/*nnc* with *a* = 1188.37(9) pm, *c* = 1232.41(9) pm) and KYb[C(CN)_3_]_4_ (*P*4/*nbm* with *a* = 1179.26(9) pm, *c* = 668.73(5) pm). Both crystal structures contain a three-dimensional framework (Niggli formula: $\begin{array}{c} 3\\ \infty \end{array}${(Yb[C(CN)_3_]_8/2_)^−^}) with Yb^3+^ in square antiprismatic coordination of terminal nitrogen atoms (*d*(Yb–N) = 241–242 pm) from eight planar star-shaped tricyanomethanide anions [C(CN)_3_]^−^. The Na^+^ or K^+^ cations occupy vacancies, which provide them with a tetrahedral coordination sphere of nitrogen (*d*(Na–N) = 239 pm vs. *d*(K–N) = 276 pm) from four [C(CN)_3_]^−^ anions. This difference results from secondary contacts with the central carbon atoms (*d*(Na–C) = 361 pm vs. *d*(K–C) = 367 pm) of four different [C(CN)_3_]^−^ units, which do not contribute to NaYb[C(CN)_3_]_4_, but effectuate a lot in the case of KYb[C(CN)_3_]_4_. The Raman spectrum recorded for NaYb[C(CN)_3_]_4_ corroborates the presence of a *pseudo-D*_3h_-symmetric tricyanomethanide anion [C(CN)_3_]^−^ and the absence of water.

## 1. Introduction

**Figure 1:**
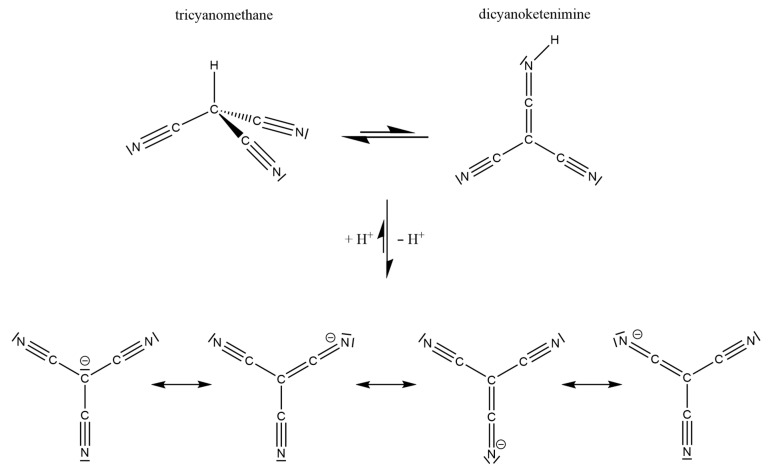
The four mesomeric resonance structures of the triangular planar tricyanomethanide anion [C(CN)_3_]^−^ (**bottom**) and its tautomeric protonation products tricyanomethane (**top**, **left**) and dicyanoketenimine (**top**, **right**), inspired by [5].

The tricyanomethanide anion [C(CN)_3_]^−^, as one of the bulkiest *pseudo-*halides, was discovered by Schmidtmann [1], but only scant research has been performed or reported for decades in spite of the chemical stability of this moiety. Therefore, some interesting features of this special anion were only recognized recently. It appears as a planar, star-shaped molecular entity (usually exhibiting *D*_3*h*_ point symmetry or something rather close to it), so its π-bonding system has to be interpreted as a delocalized 8π-electron, seven-center bond arrangement with a *sp*^2^-hybridized carbon atom in the center allowing for a participating electron pair in the *p*-orbital to carry a negative charge. According to Figure 1 (bottom), the electronic delocalization, a situation often addressed as “Y aromaticity” [2], also takes a negative charge in all three of the terminal nitrogen atoms as equally contributing mesomeric resonance structures. Upon protonation, this very stable [C(CN)_3_]^−^ anion is disturbed so much that the corresponding strong Brønsted acid H[C(CN)_3_] not only exhibits a p*K*_a_ value of −5 [3], but the synthesis and final proof for the tricyanomethane form HC(CN)_3_, which is a thermodynamically more stable tautomer as compared to the dicyanoketenimine form (NC)_2_C(CNH) (Figure 1, top), was achieved only recently [4,5]. This is despite the fact that the central carbon atom would have to change its hybridization from *sp*^2^ to *sp*^3^, as the resulting small amounts of the dicyanoketenimine tautomer reveal themselves to be very prone to oligomerization at ambient temperatures in solution. On the other hand, this dicyanoketenimine form becomes much favored when H^+^ is replaced with the “big organometallic proton” [(H_3_C)_3_Si]^+^ [5].

The anion [C(CN)_3_]^−^ usually coordinates with its terminal nitrogen atoms, especially with hard cations according to the Pearson HSAB (hard and soft acids and bases) concept [6], but a real contribution at the lone-pair electrons of the central carbon atom occurs with soft, polarizable, low-charged cations (e.g., Ag^+^ in Ag[C(CN)_3_] [7,8,9]), particularly when they also have some lone-pair activity. The unique crystal structure of Tl[C(CN)_3_] [10] with Tl^+^ as a lone-pair cation (6*s*^2^ versus 6*sp* or 6*p*^2^) compared to those with the alkali metals *A*[C(CN)_3_] (*A* = Li–Cs) [11,12,13,14,15] may serve as the best example. Even for the isotypic crystal structures of Ca[C(CN)_3_]_2_ [16,17] and Cd[C(CN)_3_]_2_ [18], there are some interesting central carbon-atom contributions.

Compounds with the trivalent rare-earth metals (*RE*) come as hydrates from aqueous solution, for instance those with the compositions *RE*[C(CN)_3_]_3_(H_2_O)_2_ (*RE* = Y and Tb–Lu), *RE*[C(CN)_3_]_3_(H_2_O)_3_ (*RE* = Sc), *RE*[C(CN)_3_]_3_(H_2_O)_4_ (*RE* = La–Nd, Sm–Tb) and *RE*[C(CN)_3_]_3_(H_2_O)_5_ (*RE* = La–Nd) [14,19]. Together with alkali metals, water-poor hydrates are formed again, e.g., KLa[(C(CN)_3_]_4_(H_2_O) [20] and K*RE*[C(CN)_3_]_4_(H_2_O) (*RE* = La–Nd, Sm–Gd) [14], but only a few representatives can be obtained in anhydrous form, namely K*RE*[C(CN)_3_]_4_ (*RE* = Tb–Lu) [14].

Since lanthanoid compounds always have potential as phosphor materials but water-containing ones often suffer from vibrational luminescence quenching, we were aiming at their *pseudo*-ternary anhydrous tricyanomethanides. The unique [C(CN)_3_]^−^ ligands with their conjugated 4c–5e π-system might even serve as an antenna for necessary energy-transfer processes. Moreover, the empirical formula K*Ln*[C(CN)_3_]_4_ with trivalent lanthanoids (*Ln*^3+^), which typically shows 4*f*–4*f* transitions, could mimic a scenario for a Eu^2+^-containing one formula unit (Eu[C(CN)_3_]_2_), where divalent europium should be able to replace both monovalent K^+^ and trivalent *Ln*^3+^ due to their similar ionic radii; much more efficient luminescence based on 4*f*–5*d* transitions leaps into view.

## 2. Methods and Experimental

### 2.1. Source of Material

**Figure 2:**
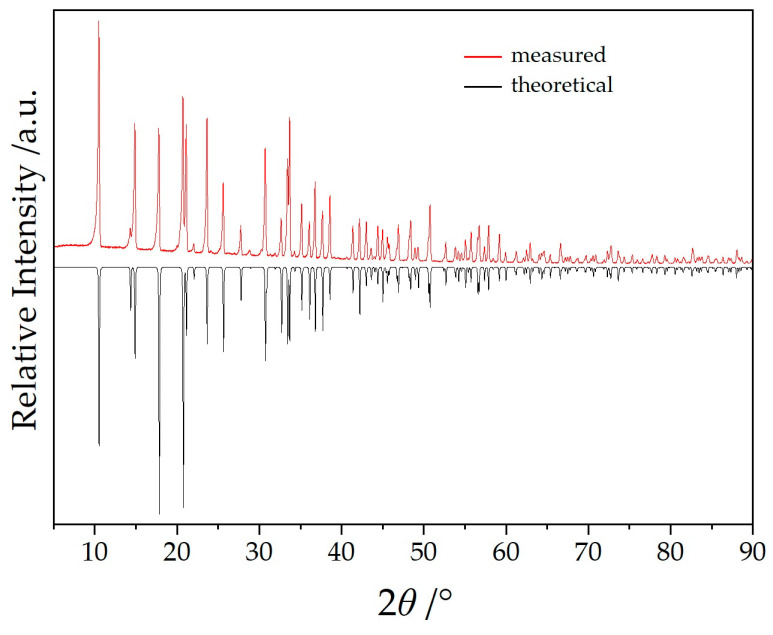
Powder X-ray diffractogram of phase-pure NaYb[C(CN)_3_]_4_.

**Figure 3:**
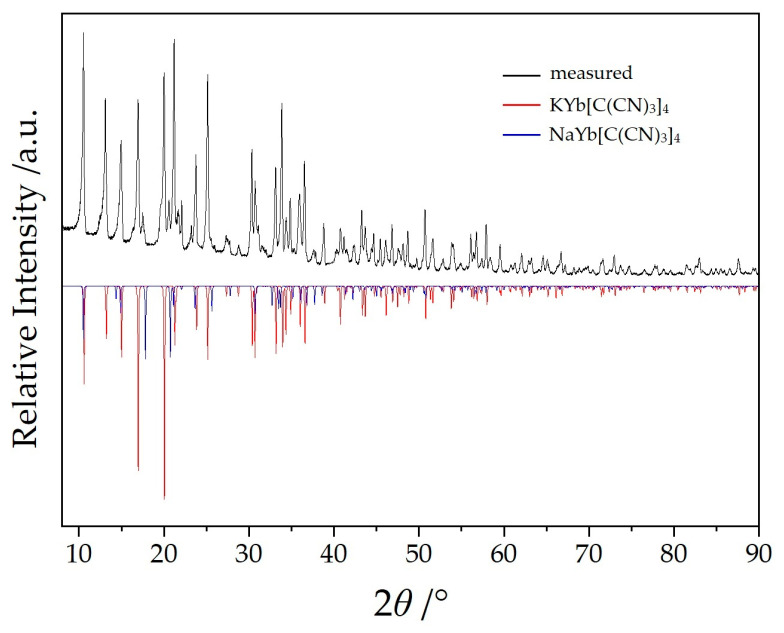
Powder X-ray diffractogram of KYb[C(CN)_3_]_4_ with NaYb[C(CN)_3_]_4_ as minority phase.

Ag[C(CN)_3_] was synthesized by blending an aqueous solution containing 1.13 g (10 mmol) of Na[C(CN)_3_] (>98%, TCI, Eschborn, Germany) with an aqueous solution of 2 g (12 mmol) Ag[NO_3_] (≥99%, Sigma-Aldrich, St. Louis, MO, USA). The resulting off-white precipitate was washed with deionized water and dried using an aspirator. A total of 340 mg (3 mmol) of Ag[C(CN)_3_] was added to 5 mL of demineralized water containing 245 mg (1 mmol) YbCl_3_ (>98%, ChemPur, Karlsruhe, Germany). To this preparation, 1 mmol of either Na[C(CN)_3_] (113 mg) or K[C(CN)_3_] (129.1 mg, 96%, powder, Alfa Aesar, Ward Hill, MA, USA) was added. After stirring the respective mixture for six hours, the resulting solution was filtered to remove the precipitated AgCl and the surplus Ag[C(CN)_3_]. The water was allowed to evaporate under ambient temperatures and atmospheric conditions. After a few days, colorless and transparent irregular crystals of the two title compounds were identified as the sole products in a quantitative yield considering the employed amount of YbCl_3_. Without the addition of *A*[C(CN)_3_] (*A* = Na or K), Yb[C(CN)_3_]_3_ crystallizes as dihydrate [14] (Yb[C(CN)_3_]_3_(H_2_O)_2_: orthorhombic, *P*2_1_2_1_2_1_; *a* = 1007.15(7) pm, *b* = 1171.57(8) pm, *c* = 1412.80(9) pm) [21]. However, when Na[C(CN)_3_] reacts with Ag[NO_3_] in aqueous slurry to form Ag[C(CN)_3_], traces of Na[C(CN)_3_] always remain, leading to the formation of NaYb[C(CN)_3_]_4_ as a by-product during the synthesis of Yb[C(CN)_3_](H_2_O)_2_. The latter can be completely converted to NaYb[C(CN)_3_]_4_ in a phase-pure manner with an excess of Na[C(CN)_3_] (Figure 2), while the product of the attempted synthesis of KYb[C(CN)_3_]_4_ always contains a share of NaYb[C(CN)_3_]_4_ (Figure 3). What is remarkable here, at first glance, is that no mixed crystals are formed in which the alkali-metal position is occupied by both sodium and potassium, but instead, despite the very similar crystal structures, both phases occur separately. On the other hand, this is in accord with the Shannon radii for Na^+^ (99 pm for *C.N.* = 4) and K^+^ (137 pm for *C.N.* = 4), so this might not come as a surprise [22].

### 2.2. Crystallographic Studies

Single-crystal selection took place with the help of a polarization microscope and suitable crystals were sealed into a thin-walled glass capillary. Intensity data sets for NaYb[C(CN)_3_] were collected with a Stoe Stadivari diffractometer (Ag-*K_α_* radiation: *λ* = 56.08 pm) and for KYb[C(CN)_3_] with a Stoe Ipds (Mo-*K_α_* radiation: *λ* = 71.07 pm). The crystallographic data are listed in Table 1 and fractional atomic coordinates and equivalent isotropic displacement coefficients are shown in Table 2. The powder samples of NaYb[C(CN)_3_]_4_ (Figure 2) and KYb[C(CN)_3_]_4_ (Figure 3) were measured in reflection geometry on a Rigaku SmartLab X-ray powder diffractometer using Cu-*K*_α1_ radiation.

### 2.3. Raman Spectroscopy

Raman spectroscopy was performed with the single crystal used for the X-ray measurements on a XploRA spectrometer (Horiba, Kyoto, Japan) with 25 mW, excitation line at *λ* = 638 nm (red LASER).

### 2.4. Thermal Analysis

The TG/DTA curves of NaYb[C(CN)_3_]_4_ and KYb[C(CN)_3_]_4_ were recorded using a Netzsch STA409 with corundum crucibles in an argon atmosphere and a heating rate of 10 °C/min^−1^.

## 3. Results and Discussion

### 3.1. Crystal Structures of NaYb[C(CN)_3_]_4_ and KYb[C(CN)_3_]_4_

**Figure 4:**
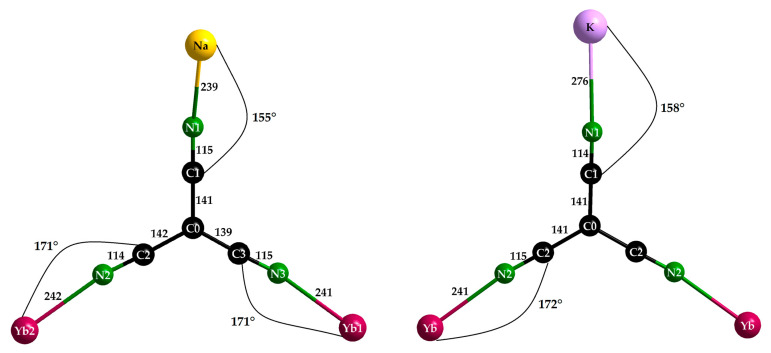
Molecular structure and coordination behavior of the [C(CN)_3_]^−^ anions in NaYb[C(CN)_3_]_4_ (**left**) and KYb[C(CN)_3_]_4_ (**right**); all distances are in pm and all angles are in °.

NaYb[C(CN)_3_]_4_ crystallizes in the tetragonal space group *P*4/*nnc* (no. 126, origin choice 2 with origin at $\bar{1}$, ^1^/_4_, ^1^/_4_, ^1^/_4_ away from 422) with the lattice parameters *a* = 1188.37 (9) and *c* = 1232.41 (9) pm (*c*/*a* = 1.037), whereas KYb[C(CN)_3_]_4_ prefers *P*4/*nbm* (no. 125, origin choice 2 with origin at 2/*m*, ^1^/_4_, ^1^/_4_, 0 away from 422) with the parameters *a* = 1179.26 (9) and *c* = 668.73 (5) pm (*c*/*a* = 0.567 = 0.5 × 1.134). Thus, for both structures, the centrosymmetric setting was chosen, since they share the same structural elements and have a surprising number of similarities. They both contain a single crystallographically independent star-shaped [C(CN)_3_]^−^ anion (Figure 4) with almost identical C–C (139–142 pm for NaYb[C(CN)_3_]_4_ and 141 pm for KYb[C(CN)_3_]_4_) and C–N distances (114–115 pm) for both (Table 3). Each anion has contact with one *A*^+^ and two Yb^3+^ cations, which graft to its three terminal nitrogen atoms (Figure 2). The Yb–N–C angles of 171° for NaYb[C(CN)_3_]_4_ and 172° for KYb[C(CN)_3_]_4_ deviate less from linearity (180°) than the Na–N–C angle of 155° and the K–N–C angle of 158°.

**Figure 5:**
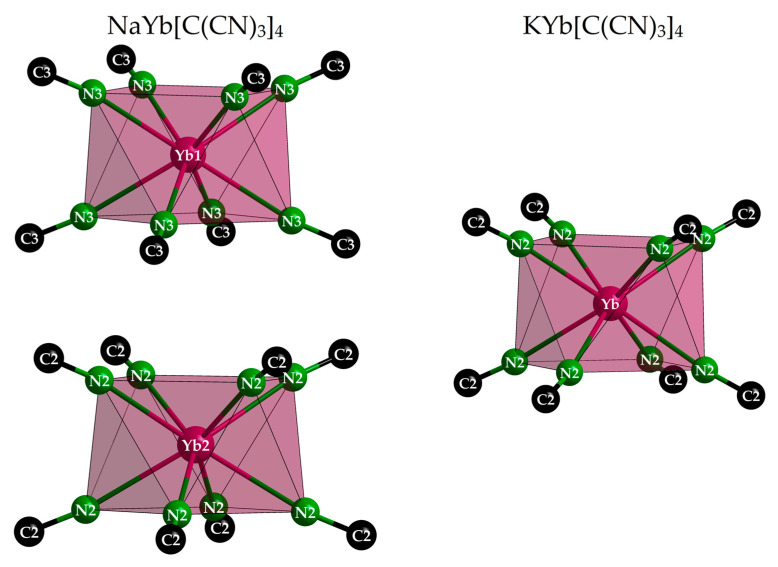
Square antiprisms [YbN_8_] with 422 symmetry in the tetragonal crystal structures of NaYb[C(CN)_3_]_4_ (**left**) and KYb[C(CN)_3_]_4_ (**right**). The [C(CN)_3_]^−^ anions are reduced to their Nn≡Cn parts for clarity reasons.

**Figure 6:**
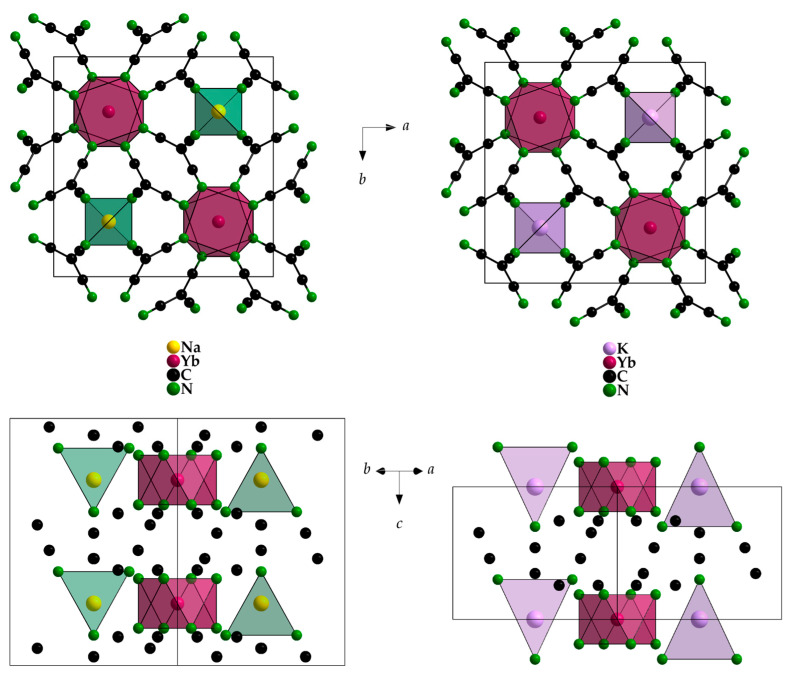
Unit-cell content of NaYb[C(CN)_3_]_4_ (**left**) and KYb[C(CN)_3_]_4_ (**right**) with views along [001] (**top**) and [110] (**bottom**).

Interestingly, in NaYb[C(CN)_3_]_4_ there are two different positions for the Yb^3+^ cations, whereas there is only one in KYb[C(CN)_3_]_4_. For magnetically interesting interactions, the shortest Yb^3+^···Yb^3+^ contacts (616 and 669 pm, respectively) seem to be much too long. However, the Yb^3+^ environment with eight nitrogen atoms from eight different [C(CN)_3_]^−^ anions as a square antiprism is almost identical (site symmetry 422 in all three cases), and even the Yb–N distances appear equal within the error tolerance at 241–242 pm (Figure 5 and Table 3). These square antiprisms [YbN_8_] form a network that can be described using the Niggli formula $\begin{array}{c} 3\\ \infty \end{array}${(Yb[C(CN)_3_]_8/2_)^−^}, with the *A*^+^ cations embedded in suitable vacancies apt to provide them [*A*N_4_] tetrahedra as first coordination spheres (Figure 6).

**Figure 7:**
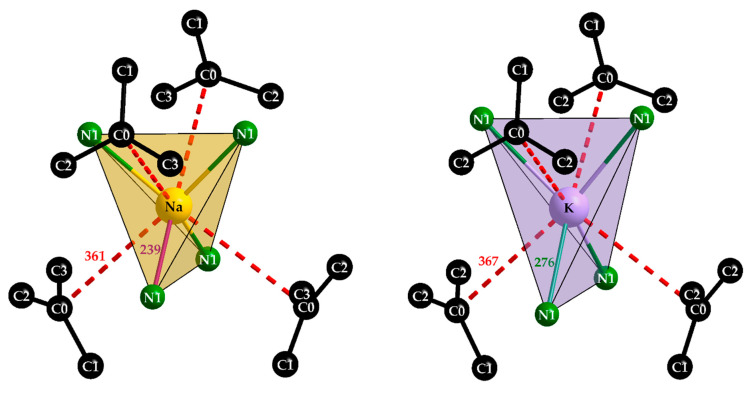
The tetrahedral environment [NaN_4_] and [KN_4_] of the alkali-metal cations Na^+^ (**left**) and K^+^ (**right**) with their secondary contacts to the carbon centers (C0) of the next unbonded [C(CN)_3_]^−^ units.

Both alkali-metal cations surround each other tetrahedrally with four [C(CN)_3_]^−^ anions and bonding takes place via the terminal nitrogen atoms again. Not surprisingly, the N–*A* distances of *d*(N–Na) = 239 pm and *d*(N–K) = 276 pm are clearly different due to the individual ionic radii of the two alkali metals (*r*_i_(Na^+^) = 100 pm, *r*_i_(K^+^) = 150 pm) [22]. This becomes more interesting with a look at the second coordination sphere (Figure 7). In each case, four further [C(CN)_3_]^−^ anions interact with the alkali-metal cations via their central C0 atoms and also form a tetrahedron around the central particles, whereby these distances are almost the same in both compounds, with a difference of 6 pm with *d*(Na···C0) = 4 × 361 pm or *d*(K···C0) = 4 × 367 pm, while there is a difference of 27 pm in their first nitrogen coordination spheres. ECoN calculations for the Effective Coordination Number [23] of both alkali-metal cations (*A*^+^) have been carried out with the following restrictions: (a) the radii for the cyanide part (Cn≡Nn) of each tricyanomethanide anion were fixed at 95 pm for Cn and Nn because of the 190 pm ionic radius of a cyanide anion ([CN]^−^) [24], (b) the radii of the involved cations were fixed at 100 pm for both Na^+^ and Yb^3+^, but at 150 pm for K^+^ [24], (c) the target was to reach an ECoN close to *one* for the N1 contribution for Na^+^ and K^+^, respectively. Astoundingly, these targets could be reached with ECoN (from N1) = 1.02 *versus* ECoN (from C0) = 0.01 for NaYb[C(CN)_3_]_4_ for *r*(C0) = 120 pm with no contribution from C0 for NaYb[C(CN)_3_]_4_, as well as ECoN (from N1) = 1.01 and ECoN (from C0) = 0.99) in the case of KYb[C(CN)_3_]_4_ for *r*(C0) = 175 pm under otherwise identical conditions. These meaningless numbers just suggest that in this case (for K^+^) the whole (C0)C_3_-star (Figure 7) acts as a ligand with its full 4c–5e π-system, what Srinivasan, Tragl and Meyer have called “a shielded potassium ion” in their KLa[C(CN)_3_]_4_ · H_2_O structure [20]. Despite a space-group change from *P*4/*n* for this hydrate (*a* ≈ 1238 pm, *c* ≈ 657 pm, *c/a* ≈ 0.53) to *P*4/*nbm* for KYb[C(CN)_3_]_4_ (*a* ≈ 1179 pm, *c* ≈ 669 pm, *c/a* ≈ 0.57) occurring, the crystal structure basically remains the same. Only the capped square antiprism around La^3+^ (*C.N.* = 9, 8 × N + 1 × O from H_2_O) turns into an uncapped one around Yb^3+^ (*C.N.* = 8, 8 × N), which is accompanied with an expected decrease in the *a*-axis due to the lanthanoid contraction, as well as an unexpected decrease in the *c*-axis, which is well reflected by the dissimilar *c/a*-values. A comparison of these naïve assessments for the NaYb[C(CN)_3_]_4_-KYb[C(CN)_3_]_4_ pair leads to the impressive result that Na^+^ (site symmetry: 222) is satisfied with its fourfold nitrogen coordination, while K^+^ (site symmetry: $\bar{4}$2m) reaches a comfortable 4 + 4 coordination with almost equal parts of N1 (4×) and C0 (4×), as examples of structure-intrinsic agostic *sp*^2^-C-*p*^2^ recognition.

The program POLYNATOR [25] verifies the shape of the polyhedra as disphenoids for Na^+^ and K^+^ on the one hand and, on the other hand, as square antiprisms for Yb^3+^ in all cases. Heterocubane-like arrangements for *C.N.* = 4 + 4 (N1)_4_(C0)_4_ around K^+^ with $\bar{4}$2*m* symmetry and Na^+^ with 222 symmetry complete the shielded coordination spheres [20] for both alkali-metal cations. Noteworthy to mention is that C0 exhibits not even the shortest contact with Na^+^ with 361 pm, since C1 comes even closer (346 pm), in contrast to the case of K^+^ (367 pm to C0 vs. 384 pm to C1).

The comparison with the *A*–N distances in Na[C(CN)_3_] (235–264 pm for *C.N.*(Na^+^) = 6) [12] and K[C(CN)_3_] (284–294 pm for *C.N.*(K^+^) = 7) [12], where no agostic *sp*^2^-C-*p*^2^ assistance is necessary to reach high coordination numbers, reveals larger values than those for both NaYb[C(CN)_3_]_4_ (239 pm, 4×) and KYb[C(CN)_3_]_4_ (276 pm, 4×), supporting the idea that there is some need for them in the latter *pseudo-*ternaries, at least for the potassium compound. The Yb–N distances in Yb[C(CN)_3_]_3_(H_2_O)_2_ (239–246 pm, 6×) [14,22] agree quite well with those (241–242 pm for *C.N.*(Yb^3+^) = 8) in both NaYb[C(CN)_3_]_4_ and KYb[C(CN)_3_]_4_, especially if one keeps in mind that two extra Yb–O contacts (227 and 228 pm) with oxygen atoms of water molecules during hydration come on top to realize *C.N.*(Yb^3+^) = 8 in the dihydrate as well.

Neglecting the light atoms carbon and nitrogen, a smaller tetragonal cell with *a’* = 841.08 (7) pm and *c’* = 616.54 (5) pm was found utilizing Mo-*K*_α_ radiation from a *κ*-CCD diffractometer from Bruker-Nonius for NaYb[C(CN)_3_]_4_. A subsequent refinement in the space group *P*4/*mmm* (no. 123) yields only one position for the Yb^3+^ and one for the Na^+^ cations, too. The light atoms carbon and nitrogen were also found and refined, but the [C(CN)_3_]^−^ units coincide in this too-small unit cell, which is related via a 1/$\sqrt{2}$ relationship to the *a*- and *b*-axes and a halved *c*-axis with the real one, and would have to be treated to induce under-occupation of these sites to avoid an eightfold coordination for Na^+^ instead of a fourfold one and a formal condensation of the discrete [C(CN)_3_]^−^ anions to fictive “(NC)_2_NC–CN(CN)_2_” units with bizarre interatomic N–C and C–C distances. Ag-*K*_α_ radiation of a STADIVARI STOE diffractometer was used to solve this problem, which led to an undoubtedly correct unit cell with $\sqrt{2}$·*a’* = $\sqrt{2}$·*b’* and 2·*c’*.

The similarity of several atomic coordinates in Table 2 (e.g., *x/a*(C2) ≈ *y/b*(C3), *x/a*(C3) ≈ *y/b*(C2), *z/c*(C2) ≈ −*z/c*(C3) and *x/a*(N2) ≈ *x/a*(N3); *y/b*(N2) ≈ *y/b*(N3); *z/c* for N2 ≈ *z/c* + ^1^/_2_ for N3) implies some higher space-group symmetry for the crystal structure of NaYb[C(CN)_3_]_4_, despite being *P*4/*nnc* already. Coding like *P*4/*n*2/*n*2/*c* → *P*4/*n*2/*b*2/*m* (2*c*’ = *c*) represents a minimal non-isomorphous supergroup of *P*4/*nbm*, and the couple NaYb[C(CN)_3_]_4_/KYb[C(CN)_3_]_4_ fulfills this requirement perfectly. Higher symmetry would either presuppose body-centering, with the inverse chloroscheelite-type arrangement of LiGdCl_4_ [26] with *C.N.*(Li^+^) = 4 and *C.N.*(Gd^3+^) = 8 standing for this analogy, in spite of the adoption of a low-Laue-symmetry space group (*I*4_1_/*a*11, no. 88), or, with preservation of the high Laue symmetry, Y[PS_4_] [27], which could serve as an example according to PYS_4_ even in the highest tetragonal space group (no. 142), namely *I*4_1_/*a*2/*c*2/*d*, which still offers two independent Y^3+^ cations in sulfur atoms with eightfold coordination from discrete [PS_4_]^3−^ tetrahedra (Y1 in 8*a* with $\bar{4}$11 symmetry and Y2 in 8*b* with 222 symmetry) [27].

### 3.2. Raman Spectrum of NaYb[C(CN)_3_]_4_

**Figure 8:**
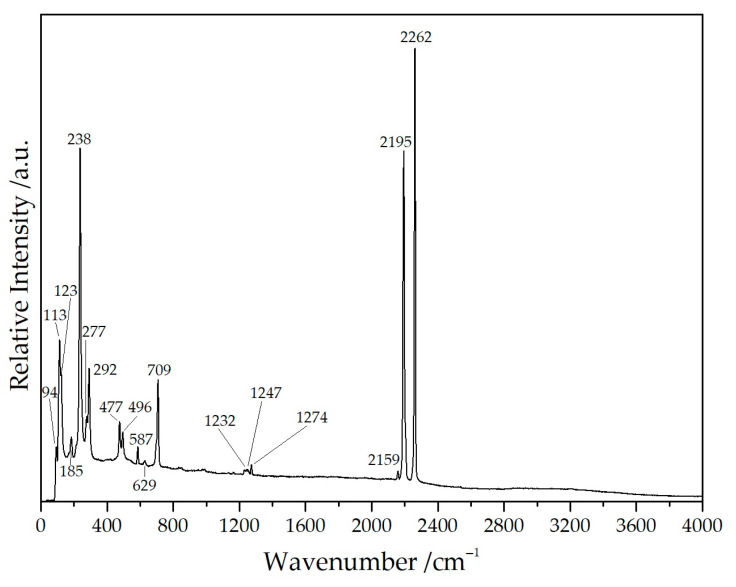
Raman spectrum of NaYb[C(CN)_3_]_4_ recorded with an excitation energy of λ = 638 nm.

The frequencies and intensities obtained from the well-resolved Raman spectrum of NaY[C(CN)_3_]_4_ (Figure 8 and Table 4) agree well with those of other salt-like tricyanomethanides with high point symmetry, e.g., Rb[C(CN)_3_] and Cs[C(CN)_3_], indicating the presence of the tricyanomethanide anion [C(CN)_3_]^−^. The splitting degree of the *ν*(C≡N) stretching mode can be taken as a good indicator for the bonding situation or the point symmetry of the tricyanomethanide anion—the higher the number of different *ν*(C≡N) stretching modes, the larger the differences in the ‘branches‘ in terms of bonding and coordination patterns exhibiting deviations from the ideal *D*_3*h*_ point symmetry. The good resolution of the spectrum even allows us to identify the weak band at 2159 cm^−1^ as the ν(^13^C≡^14^N) stretching mode [28].

Moreover, the flat region between 2400 and 4000 cm^−1^ proves the absence of any crystal-water molecules, which are present in Yb[C(CN)_3_]_3_(H_2_O)_2_ [14,21].

### 3.3. Thermal Analysis of NaYb[C(CN)_3_]_4_ and KYb[C(CN)_3_]_4_

**Figure 9:**
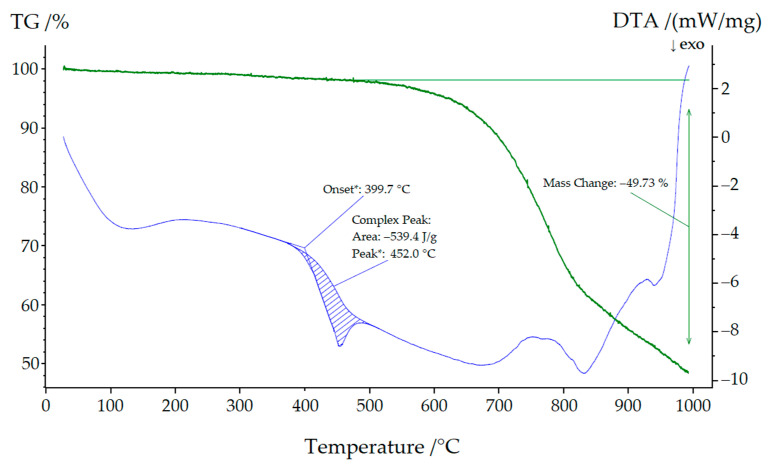
Thermal analysis of NaYb[C(CN)_3_]_4_.

**Figure 10:**
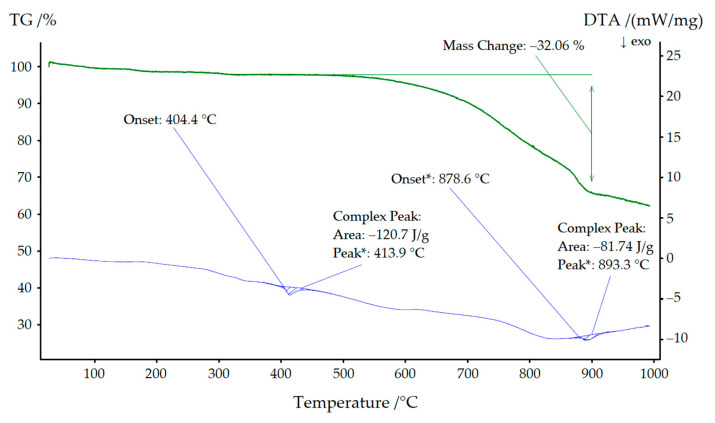
Thermal analysis of the KYb[C(CN)_3_]_4_ with NaYb[C(CN)_3_]_4_ as minority phase.

As can be seen in Figure 9, NaYb[C(CN)_3_]_4_ started to decompose at approximately 400 °C, leaving a grayish-black residue behind. Low quality powder X-ray diffractograms revealed this residue to be almost amorphous, but its black color indicated the formation of carbon. A very similar picture emerges in Figure 10, where a mixture of both compounds (KYb[C(CN)_3_]_4_ and NaYb[C(CN)_3_]_4_) was used for thermal treatment. Here, the decomposition began at 404 °C and, in addition, the mass steadily decreased, since parts of the decomposition products transitioned into the gas phase (cyanogen, (CN)_2_). We therefore assume the following decomposition reaction:

2 *A*Yb[C(CN)_3_]_4_ → 2 *A*[CN] + Yb_2_[CN_2_]_3_ + 11 C + 8 (CN)_2_ (*A* = Na and K), (1)

as we could detect at least the strongest reflections of alkali-metal cyanides (*A*[CN] [29,30]) and ytterbium(III) carbodiimide (Yb_2_[CN_2_]_3_) [31].

For KEu[C(CN)_3_]_4_(H_2_O) and KTb[C(CN)_3_]_4_, the decomposition temperatures were significantly lower (534 °C and 501 °C, respectively) [14].

Similarly, a very weak signal was detectable at 350 °C in both thermograms. A powder X-ray diffractogram of the sodium compound was of very poor quality but nevertheless showed lattice parameters (*a* ≈ 1188 pm and *c* ≈ 616 pm), which suggests that an irreversible phase transformation into the corresponding KYb[C(CN)_3_]_4_ structure occurred.

## 4. Conclusions and Outlook

By using the well-established Ag[C(CN)_3_] metathesis method, NaYb[C(CN)_3_]_4_ and KYb[C(CN)_3_]_4_ were obtained from aqueous brine. Their crystal structures are similar, but not isotypic, showing Yb^3+^ in square antiprismatic, and the alkali-metal cation *A*^+^ in disphenoidal, nitrogen coordination. Owing to the fact that the crystal structures of both NaYb[C(CN)_3_]_4_ and KYb[C(CN)_3_]_4_ show two completely different coordination geometries for the *A*^+^ and Yb^3+^ cations, it seems improbable that they could serve as plausible structural environments for a potential Eu[C(CN)_3_]_2_ arrangement describing europium(II) tricyanomethanide, where Eu^2+^ would need to replace both *A*^+^ and Yb^3+^ simultaneously. The idea to aim at anhydrous rather than hydrated *pseudo-*ternary *ALn*[C(CN)_3_]_4_ compounds seems to be proven by our preliminary finding that tetragonal NaEu[C(CN)_3_]_4_ · H_2_O (space group: *P*4/*n*) shows no luminescence at all because of quenching processes, while tetragonal NaTb[C(CN)_3_]_4_ (space group: *P*4/*nbm*) shows a strong green one. We will report on this in a separate publication in the near future.

## Figures and Tables

**Table 1:** Crystallographic data for NaYb[C(CN)_3_]_4_ and KYb[C(CN)_3_]_4_ with their determination.

*A*Yb[C(CN)_3_]_4_		*A* = Na	*A* = K
crystal system		tetragonal	
space group		*P*4/*nnc* (no. 126)	*P*4/*nbm* (no. 125)
lattice parameters,	*a*/pm	1188.37(9)	1179.26(9)
	*c*/pm	1232.41(9)	668.73(5)
	*c*/*a*	1.037 (= 2 × 0.519)	0.567
number of formula units, Z		4	2
calculated density, *D*_x_/g∙cm^−3^		2.123	2.044
unit-cell volume, *V*_uc_/Å^3^		1740.44 (2 × 870.22)	929.97
molar volume, *V*_m_/cm^3^∙mol^−1^		262.03	280.02
diffractometer		STADIVARI (Stoe & Cie)	STOE IPDS I
wavelength		Ag-*K_α_* (*λ* = 56.08 pm)	Mo-*K_α_* (*λ* = 71.07 pm)
diffractometer limit, *Θ*_max_/°		28.9	30.5
index range, ±*h*_max_, ±*k*_max_, ±*l*_max_		20, 20, 21	16, 16, 9
electron sum, *F*(000)/e^−^		1044	538
absorption coefficient, *µ*/mm^−1^		2.92	5.28
collected/unique reflections		12,198/2157	7663/764
data set residuals, *R*_int_/*R*_σ_		0.052/0.034	0.068/0.029
structure residuals, *R*_1_/*wR*_2_		0.025/0.075	0.018/0.041
goodness of fit (GooF)		0.944	0.952
residual electron density, *ρ*_max/min_/e*^−^* 10*^−^*^6^ pm*^−^*^3^		+1.35/−1.54	+0.74/−0.69
CSD number		2,455,414	2,478,707

**Table 2:** Atomic positions and equivalent isotropic displacement parameters for *A*Yb[C(CN)_3_]_4_ (*A* = Na and K).

Atom	Site	*x*/*a*	*y*/*b*	*z*/*c*	*U*_eq_/pm^2^
**NaYb[C(CN)_3_]_4_**					
Na	4*c*	^1^/_4_	^3^/_4_	^3^/_4_	236(5)
Yb1	2*a*	^1^/_4_	^1^/_4_	^1^/_4_	140(2)
Yb2	2*b*	^1^/_4_	^1^/_4_	^3^/_4_	138(2)
C0	16*k*	0.0830(6)	0.9198(6)	0.4316(6)	228(6)
C1	16*k*	0.1175(6)	0.8828(6)	0.0349(6)	239(7)
C2	16*k*	0.0142(6)	0.1320(6)	0.1145(6)	184(5)
C3	16*k*	0.1316(6)	0.0134(6)	0.8850(6)	237(7)
N1	16*k*	0.1440(6)	0.8558(6)	0.1210(6)	313(8)
N2	16*k*	0.1749(6)	0.0930(6)	0.3503(6)	285(7)
N3	16*k*	0.1744(6)	0.0918(6)	0.8503(6)	261(7)
**KYb[C(CN)_3_]_4_**					
K	2*c*	^1^/_4_	^3^/_4_	0	300(3)
Yb	2*a*	^1^/_4_	^1^/_4_	0	132(1)
C0	8*m*	0.0792(2)	–0.0792(2) ^a)^	0.3462(5)	202(7)
C1	8*m*	0.1118(2)	–0.1118(2) ^a)^	0.5402(5)	238(9)
C2	16*n*	0.1311(2)	0.0153(2)	0.2568(4)	201(5)
N1	8*m*	0.1367(2)	–0.1367(2) ^a)^	0.7001(5)	350(8)
N2	16*n*	0.1746(2)	0.0924(2)	0.1864(4)	275(5)

^a)^ ≡ −*x/a* by space-group symmetry.

**Table 3:** Selected interatomic distances (*d*/pm) for NaYb[C(CN)_3_]_4_ (**left**) and KYb[C(CN)_3_]_4_ (**right**).

NaYb[C(CN)_3_]_4_			KYb[C(CN)_3_]_4_		
Na–N1	4×	238.7(3)	K–N1	4×	275.5(3)
Na···C0	4×	360.8(4)	K···C0	4×	366.8(5)
Yb1–N2	8×	240.9(2)	Yb–N2	8×	240.8(2)
Yb2–N3	8×	242.3(2)			
C0–C1	1×	140.8(4)	C0–C1	1×	140.7(5)
C0–C2	1×	138.6(5)	C0–C2	2×	140.5(3)
C0–C3	1×	142.0(5)			
C1–N1	1×	115.2(4)	C1–N1	1×	114.7(5)
C2–N2	1×	115.1(5)	C2–N2	1×	114.6(3)
C3–N3	1×	114.4(5)			

**Table 4:** Raman data for NaYb[C(CN)_3_]_4_ and Cs[C(CN)_3_] [15] as reference; wavenumbers are given in cm^−1^ (intensity coding: vs = very strong, s = strong, m = medium, w = weak, vw = very weak, sh = shoulder).

	NaYb[C(CN)_3_]_4_	Cs[C(CN)_3_] [15]
Lattice vibrations	94 sh/113 m/123 sh 185 w/238 s/277 sh/292 m	89 s/194 s
δ(C–C_3_) i.p.	477 w/496 w	484 vw
ν(C–C≡N) i.p.	587 w/629 vw	–
ν(C–C)	709 m	648 m
*δ*(C–C)	–	972 vw
ν(C–C)	1232 vvw/1247 vvw/1274 vw	1232 vw/1265 vw
ν(^13^C≡^14^N)	2159 w	2129 vw
ν(^12^C≡^14^N)	2195 s/2262 vs	2175 vs/2220 vs

## Data Availability

Data are available from the authors upon request.
